# Microencapsulation of Propolis and Honey Using Mixtures of Maltodextrin/Tara Gum and Modified Native Potato Starch/Tara Gum

**DOI:** 10.3390/foods12091873

**Published:** 2023-04-30

**Authors:** Carlos A. Ligarda-Samanez, David Choque-Quispe, Elibet Moscoso-Moscoso, Mary L. Huamán-Carrión, Betsy S. Ramos-Pacheco, Germán De la Cruz, José C. Arévalo-Quijano, Jenny C. Muñoz-Saenz, Mauricio Muñoz-Melgarejo, Uriel R. Quispe-Quezada, Edgar Gutiérrez-Gómez, Rober Luciano-Alipio, Miluska M. Zamalloa-Puma, Genaro Julio Álvarez-López, Reynaldo Sucari-León

**Affiliations:** 1 Food Nanotechnology Research Laboratory, Universidad Nacional José María Arguedas, Andahuaylas 03701, Peru; 2 Nutraceuticals and Biomaterials Research Group, Universidad Nacional José María Arguedas, Andahuaylas 03701, Peru; 3 Research Group in the Development of Advanced Materials for Water and Food Treatment, Universidad Nacional José María Arguedas, Andahuaylas 03701, Peru; 4 Agroindustrial Engineering, Universidad Nacional José María Arguedas, Andahuaylas 03701, Peru; 5 Water Analysis and Control Research Laboratory, Universidad Nacional José María Arguedas, Andahuaylas 03701, Peru; 6 Agricultural Science Faculty, Universidad Nacional de San Cristobal de Huamanga, Ayacucho 05000, Peru; 7 Department of Education and Humanities, Universidad Nacional José María Arguedas, Andahuaylas 03701, Peru; 8 Human Medicine Faculty, Universidad Peruana los Andes, Huancayo 12006, Peru; 9 Agricultural and Forestry Business Engineering, Universidad Nacional Autónoma de Huanta, Ayacucho 05000, Peru; 10 Engineering and Management Faculty, Universidad Nacional Autónoma de Huanta, Ayacucho 05000, Peru; 11 Administrative Sciences Faculty, Universidad Nacional Autónoma Altoandina de Tarma, Junín 12731, Peru; 12 Department of Physics, Universidad Nacional de San Antonio Abad del Cusco, Cusco 08000, Peru; 13 Law and Humanities Faculty, Universidad Continental, Cusco 08000, Peru

**Keywords:** microencapsulation, spray drying, ethanolic extract of propolis, bee honey, maltodextrin, modified starch, tara gum, bioactive compounds

## Abstract

Ethanolic extracts of propolis and bee honey contain substances beneficial to human health. Mixtures of wall materials were compared in spray-drying microencapsulation of ethanolic extracts of propolis and bee honey rich in bioactive compounds. Maltodextrin and tara gum were used to obtain microencapsulates A, and modified native potato starch and tara gum were used for microencapsulates B. High values of phenolic compounds, flavonoids, and antioxidant capacity were obtained in microcapsules A and B, and the results obtained in terms of encapsulation efficiency, yield, hygroscopicity, solubility, moisture, Aw, bulk density, and color were typical of the spray-drying process. On the other hand, spherical and elliptical microparticles of sizes between 7.83 and 53.7 µm with light and medium stability were observed. Thermogravimetric properties were similar in both microencapsulates; total organic carbon, SEM-EDS, and FTIR analyses corroborated the encapsulation. X-ray diffractogram exhibited amorphous structures, and the release kinetics of phenolic compounds presented high values from 8.13 to 12.58 mg GAE/g between 7 and 13 h. Finally, modified potato starch is a better encapsulant than maltodextrin because it has better core protection and controlled release of the encapsulated bioactive compounds.

## 1. Introduction

Propolis contains substances elaborated by bees (*Apis Mellifera L.*), with significant antioxidant and preservative properties, which can be used in the production of food and pharmaceutical products. However, its use is scarce due to its bitter taste and insolubility in water [1,2,3]. Honey is also a raw material produced by bees, valued for its flavor, aroma, and health-promoting properties; it is a food accepted by consumers, which is why it is added to various products [4,5]. It is known that bioactive compounds in honey contribute to the functional properties of foods [6].

Microencapsulation is a method that improves the properties of the spray-dried core, taking into account the matrices used and the operating conditions of the equipment. In addition, it facilitates the handling of bioactive compounds, improves their solubility and stability, protects the degradation of the cores, controls the release of compounds, and masks unpleasant tastes and odors [1,7]. Microcapsules obtained by this technique can be mononuclear or polynuclear; the wall material coating the active ingredient can present more than one layer [8]. The differences in microencapsulation are not only due to the encapsulants used but also to the interactions they have with the cores; the use of mixtures in both cores and matrices is an alternative to improve encapsulation processes, so a field to explore in the future is the use of new biopolymers, formulations of mixtures, and their characterization [9].

Maltodextrin is obtained from the hydrolysis of starches, is water-soluble, tasteless, and of low viscosity, and is considered a good encapsulant that can be acquired at economical prices [10,11,12]. Tara gum (*Caesalpinia spinosa)* is a galactomannan used as a wall material obtained by drying and has a chain made up of β-D-mannose units [13,14]. Native potato starches are used in the food industry because of their advantages in gel formation and low retrogradation [14,15]. From native starch, octenyl succinylated starch can be obtained, frequently used as an encapsulating agent in food and pharmaceutical products [16].

There are a limited number of natural biopolymers available as encapsulation matrices, the use of modified gums and starches are considered favorable for spray drying, so it is necessary to evaluate their behavior due to insufficient knowledge about their compatibility with the core and other physical, chemical, and structural properties [17]. New biopolymers are considered in the microencapsulation of various bioactive compounds; there are recent investigations about spherical starch aggregates and their possible future applications [18], so in the present study, the use of unconventional wall materials, obtained from alternative plant sources from the Andean region of Peru, which offer comparative advantages of protection and release of encapsulated bioactive compounds, were compared. Finally, the research aimed to microencapsulate honey and ethanolic extracts of propolis, using combinations of maltodextrin, tara gum, and modified native potato starch as protective matrices.

## 2. Materials and Methods

### 2.1. Materials

**Figure 1:**
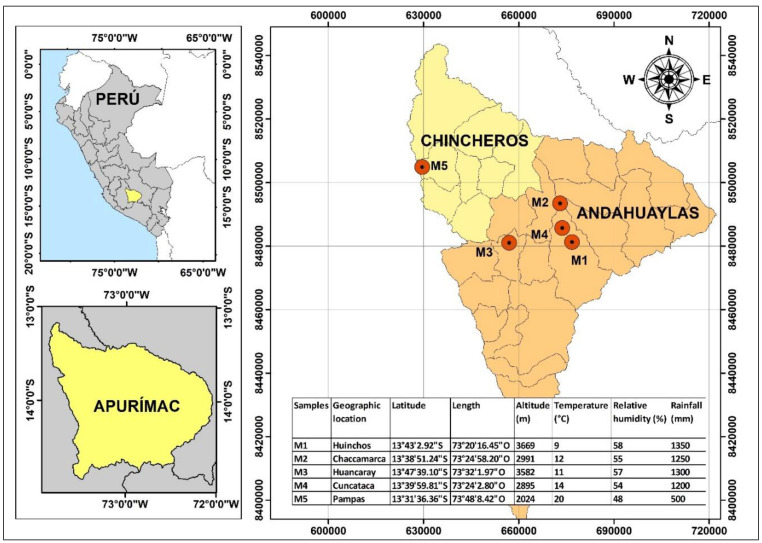
The geographical location of propolis and honey sample collection sites.

Samples were collected in Huinchos (M1), Chaccamarca (M2), Huancaray (M3), Cuncataca (M4), and Pampas (M5), as shown in Figure 1. The native potato of the “*peruanita*” variety was obtained from farmers in the district of San Jeronimo in Apurimac Peru. Once the raw materials were collected, they were stored in closed jars and refrigerated until their use. In the case of tara gum, a commercial organic sample was acquired, produced by “*Molinos Asociados S.A.C.”* under the brand name “*mama tara*,” used as a gluten free natural thickener for food use.

### 2.2. Obtaining Native Potato Starch Modified with Octenyl Succinic Anhydride (OSA)

A total of 45 g of native potato starch (previously obtained by hydro extraction [19]) was dispersed in 100 mL of distilled water. The pH of the solution was adjusted to 8.5 with 0.1 M NaOH, and then 3% octenyl succinic anhydride was added. After 6 h, the pH was adjusted to 7 using citric acid. Subsequently, it was washed three times with distilled water and centrifuged (TDL-5M centrifuge, Bioridge, Shangai, China) at 3000 rpm for 10 min for a final wash with 96% ethanol. Finally, the OSA starch was dried (convection oven FED 115, Binder, Tuttlingen, Germany) at 40 °C for 12 h and sieved using the 63 µm mesh in an analytical sieve shaker (AS 200 model, Retsch, Haan, Germany) [20].

### 2.3. Obtaining Ethanolic Extracts from Propolis

**Figure 2:**
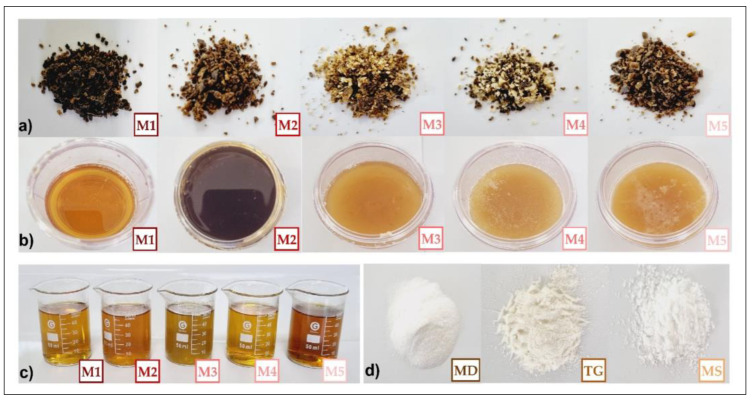
(**a**) Crude propolis, (**b**) honeys, (**c**) ethanolic extracts of propolis, and (**d**) maltodextrin (MD), tara gum (TG), and modified starch (MS).

The crude propolis samples (15 g) were shaken with 0.1 L of 80% ethanol for 24 h and filtered through a 25 µm filter. Subsequently, the supernatants were obtained by centrifugation at 4500 rpm for 10 min and 5 °C and stored at 4 °C until use [2]. Figure 2a shows crude propolis samples, Figure 2b honey samples, Figure 2c ethanolic extracts of propolis, and Figure 2d encapsulants used.

### 2.4. Microencapsulation of Ethanolic Extracts of Propolis and Bee Honey

**Figure 3:**
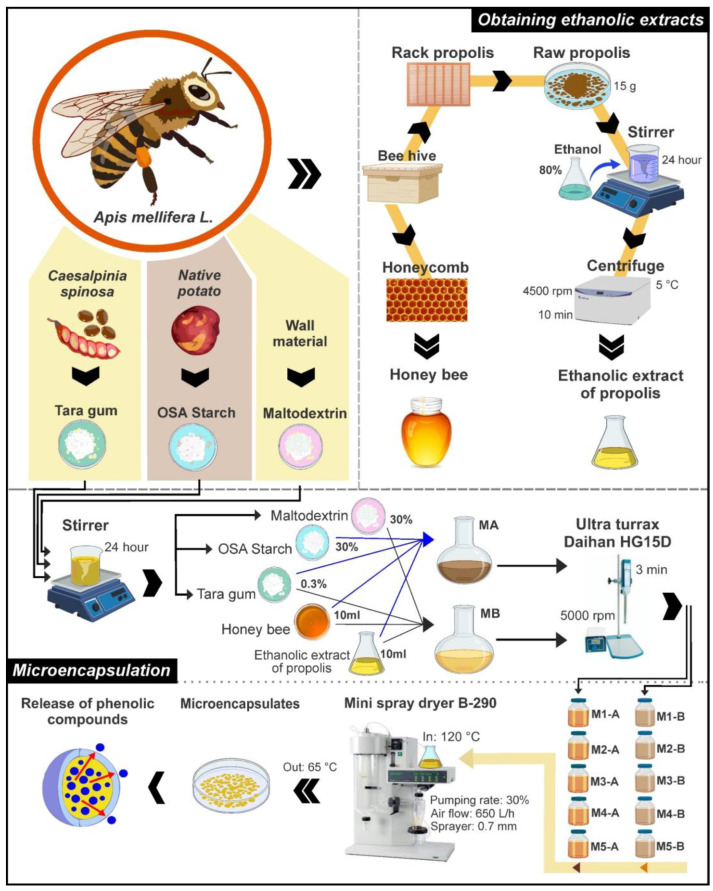
Experimental flow diagram.

Figure 3 shows the detailed experimental diagram; to obtain microencapsulates A, 10 mL of extract and 10 mL of honey were mixed, using 30% maltodextrin and 0.3% tara gum (*w*/*v*) as an encapsulant. In the case of microencapsulates B, the same proportion of extract and honey was mixed, but using 30% OSA starch and 0.3% tara gum (*w*/*v*) as an encapsulant.

The wall materials were left in agitation for 24 h using a thermo-magnetic shaker (M6 model, CAT, Ballrechten-Dottingen, Germany); to add the mixtures of extracts and honey, they were homogenized at 5000 RPM for 3 min in an Ultra Turrax (Daihan, HG15D, Wonju, Korea). For spray drying (Mini Spray Dryer B-290, Büchi Labortechnik AG, Flawil, Switzerland) was used a temperature of 120 °C, air flow of 650 L/h, and 0.7 mm sprayer. Microencapsulates were stored in airtight polyethylene containers in a desiccator until analysis [14].

### 2.5. Total Flavonoids Content

The extract was mixed with methanol and aluminum chloride, leaving the mixture in darkness for 10 min. Subsequently, the samples were read at 450 nm on a spectrophotometer (Genesys 150, Thermo Fisher Scientific, Waltham, MA, USA) using a quercetin calibration curve in the range from 0.2 to 1.2 mg/mL (R^2^ of 0.97) [2,21]. This analysis was performed on crude propolis, ethanolic propolis extracts, honey, and microencapsulates. Results were expressed on a dry basis as mg quercetin per g of sample.

### 2.6. Total Phenolic Compounds Content

The extract was mixed with 20% sodium carbonate, 0.25 N Folin Ciocalteau’s reagent, and ultrapure water. Subsequently, the samples were read at 755 nm on a spectrophotometer using a gallic acid calibration curve in the range from 5 to 35 mg/L (R^2^ of 0.98) [2,22]. This analysis was performed on crude propolis, ethanolic propolis extracts, honey, and microencapsulates. Results were expressed on a dry basis as mg gallic acid equivalent (GAE) per g of sample.

### 2.7. Antioxidant Capacity Obtained by 2,2 Diphenyl-1-picrylhydrazyl (DPPH) and 2,2’-azinobis-3-ethylbenzothiazoline-6-sulfonic Acid (ABTS) Methods

For the DPPH method, 1.25 mM DPPH stock solution was diluted with 80% methanol to an absorbance reading of 1.1 ± 0.02 at a wavelength of 515 nm. The extract was mixed with diluted DPPH solution; after 10 min at room temperature, readings were taken at 515 nm on a spectrophotometer using a Trolox reagent calibration curve in the range from 10 to 699.19 µmol/L (R^2^ of 0.98) [23,24]. Results were expressed on a dry basis as µmol of Trolox per g of sample.

For the ABTS method, 300 µL of extract was mixed with the previously prepared 2.7 mL ABTS+ radical solution (2.45 mM potassium persulfate solution was mixed with 7 mM ABTS solution (1:1, *v*/*v*) to react 8 h at the room temperature in dark); after 15 min at room temperature, readings were taken at 734 nm on a spectrophotometer using a Trolox reagent calibration curve in the range from 10 to 300 µmol/L (R^2^ of 0.97) [25,26]. Results were expressed on a dry basis as µmol of Trolox per g of sample.

Both analyses were performed on crude propolis, ethanolic propolis extracts, honey, and microencapsulates.

### 2.8. pH and Soluble Solids in Honey

The pH of the honey was determined using a Lab 885 potentiometer (SI Analytics, Mainz Germany), previously calibrated. The soluble solids of the samples were determined with an Abbe AR12 table refractometer (Schmidt Haensch, Germany) at a temperature of 20 °C. The results were expressed in °Brix.

### 2.9. Encapsulation Efficiency, Yield, Hygroscopicity, and Solubility in Microencapsulates

Encapsulation efficiency was determined based on the content of total phenolic compounds [2,27]. A total of 0.5 g of microencapsulates was dissolved in 20 mL of 80% methanol, then centrifuged at 3000 RPM for 15 min to obtain the supernatant. Next, readings were taken at 755 nm using a calibration curve of gallic acid (R^2^ of 0.98) and the following formula was used.

(1) $$EE\left(\%\right)=\frac{PCe}{PCi}\cdot 100$$

where *EE* is encapsulation efficiency (%), *PCe* is the content of total phenolic compounds in the microencapsulates, and *PCi* is the total phenolic content of the core (mixture of ethanolic extracts of propolis and honey).

The encapsulation yield was measured by relating the final mass of the microencapsulates to the initial mass of the core and wall materials using the following formula [2].

(2) $$Y=(\frac{m_{1}}{m_{2}})\cdot 100$$

where Y is encapsulation yield (%), m_1_ is initial mass of the core and wall materials (g), and m_2_ is final mass of microencapsulates (g).

To determine hygroscopicity, an open petri dish with 1 g of microencapsulates was set up inside an airtight container with saturated NaCl solution, leaving the samples for seven days at 25 °C. Subsequently, the final mass was noted and calculated using the following formula [2].

(3) $$H=(\frac{m_{3}-m_{2}}{m_{2}-m_{1}})\cdot 100$$

where H is the hygroscopicity (%), m_1_ is the mass of the empty Petri dish, m_2_ is the mass of the petri + microencapsulates, and m_3_ is the mass of the petri + microencapsulates after seven days.

Finally, 2.5 g of microencapsulate was dissolved in 0.25 L of water to determine the solubility; the solutions were stirred with a vortex mixer for 5 min at room temperature. The supernatant was separated by centrifugation (TDL-5M centrifuge, Bioridge, Shanghai, China) for 5 min at 5000 RPM. The supernatants were dried (FED 115 oven, Binder, Tuttlingen, Germany) at 105 °C for 5 h. Solubility was calculated according to the following formula [2].

(4) $$S=(\frac{m_{2}}{m_{1}})$$

where S is the solubility (%), m_1_ is the initial mass of the microencapsulates, and m_2_ is the final mass after drying.

### 2.10. Moisture, Water Activity (Aw), Bulk Density, and Color in Microencapsulates

The percentage of moisture was determined by drying in an oven method [28], and Aw was quantified instrumentally with a HygroPalm23-AW (Rotronic brand, Bassersdorf, Switzerland). The bulk density was calculated considering the mass in grams of microencapsulates and the final volume, for which the samples were placed in 10 mL test tubes that were shaken on a flat surface [2]. Color determination was carried out with a colorimeter (model CR-5, Konica Minolta, Tokyo, Japan), in which chroma a*, chroma b*, and lightness L* were measured.

### 2.11. Analysis of Total Organic Carbon (TOC) in Microencapsulates

A total of 50 mg of microcapsules were weighed into ceramic containers and measured in a TOC-L CSN-SSM 5000th total organic carbon analyzer (Shimadzu, Kyoto, Japan) [2].

### 2.12. Analysis by Scanning Electron Microscopy (SEM) and Energy Dispersive X-ray Spectroscopy (EDS)

A Prism E SEM (Thermo Fisher, Waltham, MA, USA) was used for morphological and elemental surface analysis. The microencapsulates were placed on a carbon tape and measured at 20 kV with magnifications of 2000× and 600× [2]. SEM analysis was also performed on maltodextrin, tara gum, and modified starch.

### 2.13. Analysis of Particle Size and Potential ζ

Particle size was measured by diffraction of light from a 600 nm Helium-Neon (He-Ne) laser on a Mastersizer 3000 (Malvern Instruments, Worcestershire, UK). The microencapsulates in a sufficient amount were dispersed in isopropanol and sonicated for 60 s until optimal obscuration was reached in the equipment. The results were obtained from the average of ten measurements per sample. The potential ζ was measured on a Zetasizer ZSU3100 (Malvern Instruments, Worcestershire, UK) equipped with a He-Ne laser at a wavelength of 632.8 nm. Microencapsulates were dispersed in ultrapure water and sonicated for 60 s. Readings were taken at 25 °C for five measurements for each sample; the DTS1080 disposable folded capillary cell was used. The potential ζ is a measure varying between ± 100 mV, which provides insight into the stability of the particles as a function of their surface charge [29].

Particle size and potential ζ analysis were also performed on maltodextrin, tara gum, and modified starch.

### 2.14. Thermal Analysis in Microencapsulates

For thermogravimetric analysis (TGA) and differential thermal analysis (DTA), a TGA 550 thermal analyzer (TA Instrument, New Castle, DE, USA) was used, for which 10 g of microencapsulates was weighed and then heated at 10 °C per minute on a ramp between 20–600 °C in the presence of nitrogen gas [2].

### 2.15. Analysis by Fourier Transform Infrared Spectroscopy (FTIR)

For the determination of the chemical groups reported in the present study, Nicolet IS50 FTIR equipment (ThermoFisher, Waltham, MA, USA) was used, making use of the transmission module in the middle range of the IR spectrum (400–4000 cm^−1^); it was prepared tablets with 99% potassium bromide and 1% sample [2]. Analyses were performed on wall materials, crude propolis, honey, ethanolic extracts of propolis, and microencapsulates.

### 2.16. Analysis by X-ray Diffraction (XRD)

The microencapsulates were packed in airtight sample holders and then analyzed in a Bruker diffractometer, model D8-Focus (Karlsruhe, Germany) [2].

### 2.17. Kinetic Study of Phenolic Compounds

Aqueous solutions of 0.01 g/mL of the microencapsulates were prepared and stored at 20 °C in the absence of light for 0, 6, 24, and 48 h, after which readings were taken at a wavelength of 755 nm in a spectrophotometer. To determine the release kinetics of phenolic compounds influenced by pH, the pH of the aqueous solutions was regulated to 3, 4, 5, and 6 with citric acid buffer solutions and 0.1 M sodium citrate dehydrate [2,11,22].

### 2.18. Particle Size in Aqueous Solution

In order to understand the stability of microencapsulates in aqueous phase products, particle size was measured in a Zetasizer ZSU3100 (Malvern Instruments, Worcestershire, UK) equipped with a He-Ne laser at a wavelength of 632.8 nm. The microencapsulates were dispersed in water and shaken for 60 sec. Readings were taken at 25 °C, with five measurements for each sample using the DTS002 cell.

### 2.19. Statistical Analysis

Significant differences were determined using Tukey’s multiple range test with a 95% confidence level, and an analysis of variance was performed beforehand. The Origin Pro 2023 program (OriginLab Corporation, Northampton, MA, USA) was used for the graphical representation and statistical tests.

## 3. Results and Discussion

### 3.1. Characterization of Crude Propolis and Ethanolic Propolis Extracts before Microencapsulation

**Figure 4:**
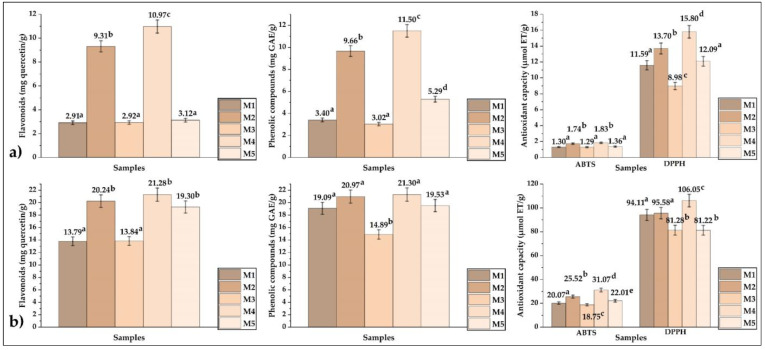
(**a**) Content of flavonoids, polyphenols, and antioxidant capacity in crude propolis, (**b**) content of flavonoids, polyphenols, and antioxidant capacity in ethanolic extracts of propolis.

The values of total polyphenols, total flavonoids, and antioxidant capacity (DPPH and ABTS) in the raw propolis are shown in Figure 4a, in which it could be seen that the sample with the highest levels of the properties studied was M4. Figure 4b shows the same analyses performed on ethanolic extracts of propolis; the M4 sample also obtained the best values, noting that the extraction operation by maceration increased the levels of the properties studied. Similar results were obtained using maceration and ultrasound methods in other investigations [30,31,32,33,34].

### 3.2. Characterization of Samples of Honey before Microencapsulation

**Figure 5:**
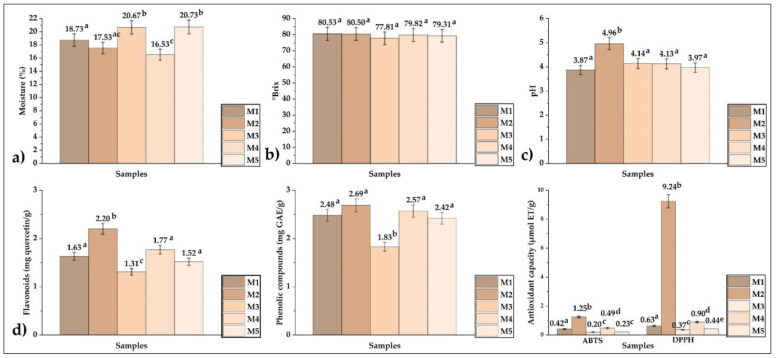
(**a**) Moisture, (**b**) soluble solids, (**c**) pH, (**d**) flavonoid content, phenolic compounds, and antioxidant capacity in honey samples.

Moisture content ranged from 16.53 to 20.67% (Figure 5a), soluble solids ranged from 77.81 to 80.53 °Brix (Figure 5b), and pH values ranged from 3.87 to 4.96 (Figure 5c); these results are typical for honey samples [35]. Figure 5d shows the results of total flavonoids (1.31–2.20 mg quercetin/g), total polyphenols (1.83–2.69 mg GAE/g), and antioxidant capacity DPPH (0.37–9.24 µmol ET/g) and ABTS (0.20–1.25 µmol ET/g); it was observed that sample M2 obtained the highest values in the properties studied.

### 3.3. Characterization of Wall Materials before Microencapsulation

**Figure 6:**
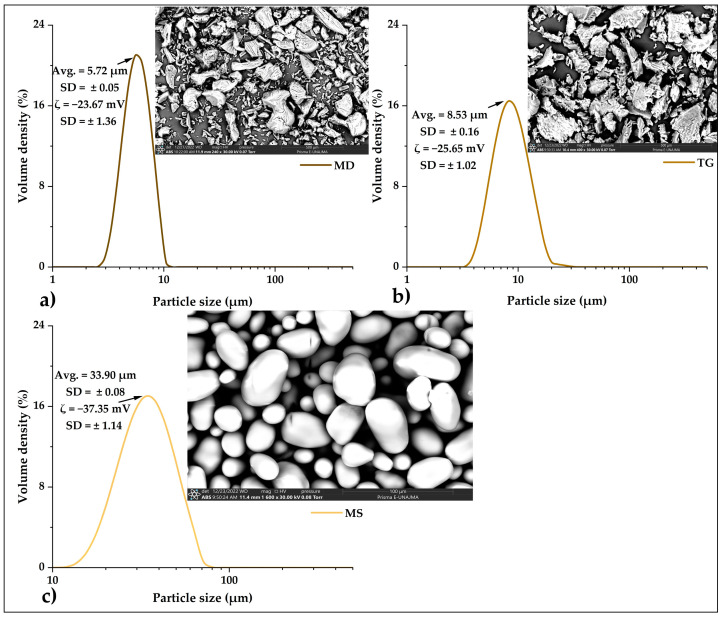
SEM micrographs, laser diffraction particle size, and potential ζ of (**a**) maltodextrin (MD), (**b**) tara gum (TG), and (**c**) OSA starch (MS).

Figure 6a shows the properties studied in maltodextrin; the SEM microphotography shows heterogeneous particles with a size of 5.72 µm and potential ζ of −23.67 mV, which indicates that these particles have slight stability. In Figure 6b, the same properties are observed in the tara gum, the microphotograph shows heterogeneous particles with a size of 8.53 µm, and the potential ζ value of −25.65 mV indicates slight stability. Finally, Figure 6c shows the properties studied in OSA starch; the microphotograph shows homogeneous particles with a size of 33.90 µm, and the potential ζ value of −37.35 mV indicates this starch has medium stability in the solution.

### 3.4. Characterization of Microencapsulates

#### 3.4.1. Physical and Chemical Properties of Microencapsulates

Table 1 shows all the results obtained; in the case of phenolic compounds and flavonoids, it was observed that the microencapsulated M4-A and M4-B reported the highest values and something similar occurred with their respective antioxidant capacities (DPPH and ABTS). Observing a direct proportional relationship between bioactive compounds and antioxidant capacity [11,36], it could also be seen that the maltodextrin/tara gum mixture allowed obtaining higher values. This is attributed to the fact that the wall materials also contribute to the functional properties studied, in addition to the honey cores and ethanolic propolis extracts incorporated [2,37].

The encapsulation efficiency was high for microencapsulates A and B, especially for samples M4-A and M4-B. All the results obtained were above the values reported for spray-dried propolis encapsulates in gum arabic and maltodextrin [2,11]. Encapsulation efficiency is affected by the encapsulation matrices and their interactions with phenolic compounds, flavonoids, and spray dryer inlet temperature [38]. Encapsulation yields were below 60%, a limit value in spray drying processes [2,37]. The values of hygroscopicity in microencapsulates A and B were similar to those reported in propolis encapsulates, which were around 8% [2,37], with 20% considered as the limit value for adequate preservation of dehydrated products [39,40].

The solubility in microencapsulates A varied between 89.63 and 92.84% and was higher than that of microencapsulates B, which ranged between 76.14 and 82.89%. The fact that spray drying was carried out at a temperature of 120 °C favored obtaining these high values. Lower 45.27–61.29% results were reported for propolis encapsulated by vacuum drying [36]. Solubility is considered an essential property in microencapsulates, as it provides insight into how they might behave when incorporated as additives in aqueous phase foods [41]; the inlet temperature of the spray drying equipment used affects solubility [42,43,44].

The moisture of the microencapsulates was around 6%, and lower values of between 1.64 and 2.21% were reported in the microencapsulation of purified propolis [37]; it is recommended that the moisture should be below 5% for the conservation of spray-dried products [45,46,47]. It is recommended that water activity values be below 0.6; in the present study, lower values were observed in microencapsulates A and B, which is essential to keep reactions linked to enzymatic and non-enzymatic browning under control [48,49]. The bulk density varied between 0.31 and 0.41, noting that higher results were obtained at smaller particle sizes [47,50]. All microencapsulates were white with lightness L* values between 90.81 and 92.82. The inlet temperature and the proportion of wall materials used influenced the color of spray-dried microencapsulates [36,51]; and color is an important parameter in food selection [36].

The principal component analysis (PCA) is a statistical technique that describes a set of factors, reducing the dimensionality of the data and allowing appreciation of the relationship between many complex variables [2,14,52,53]. A PCA was performed for the results obtained for microencapsulates A and B, in which a positive correlation was observed.

**Figure 7:**
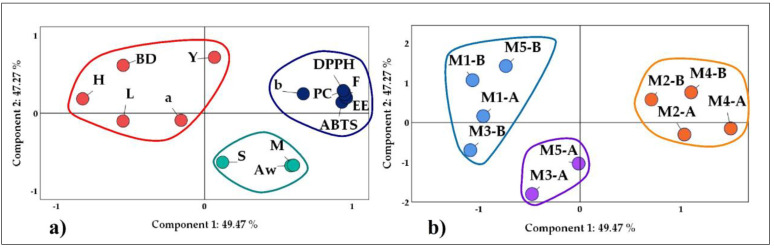
PCA analysis: (**a**) properties studied and (**b**) microencapsulates A and B.

The first group in blue (Figure 7a) was formed by phenolic compounds (PC), flavonoids (F), DPPH and ABTS antioxidant capacity, encapsulation efficiency (EE), and the color parameter b, variables mostly related to microencapsulates M2-A, M2-B, M4-A, and M4-B (in orange Figure 7b).

The second group in red (Figure 7a) was formed by yield (Y), hygroscopicity (H), bulk density (BD), and the color parameters a and L, preferentially related to microencapsulates M1-A, M1-B, M3-B, and M5-B (in light blue Figure 7b).

The last group in green (Figure 7a) was formed by solubility (S), moisture (M), and Aw associated with the microencapsulates M3-A and M5-A (in purple Figure 7b).

#### 3.4.2. Analysis of Total Organic Carbon (TOC)

**Figure 8:**
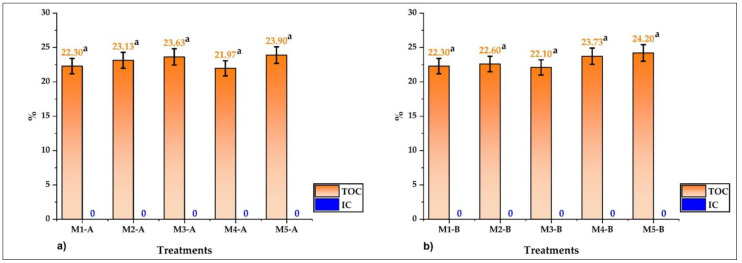
(**a**) TOC and inorganic carbon (IC) in microencapsulates A and (**b**) TOC and IC in microencapsulates B.

Microencapsulates A presented values between 21.97 and 23.90% (Figure 8a); regarding microencapsulates B, values between 22.10 and 24.20% were reported (Figure 8b). No inorganic carbon was reported in both cases, and no significant differences were found (*p* > 5%). These results indicate that these are all organic samples containing glucides, proteins, lipids, and fiber, components that are part of the propolis, honey, and wall materials used [2,7,54,55]. The total organic carbon values correlate with the surface elemental analysis performed in this study, which corroborates the presence of organic compounds in microencapsulates A and B obtained by spray drying [2,10,56,57,58].

#### 3.4.3. Analysis by SEM and EDS

**Figure 9:**
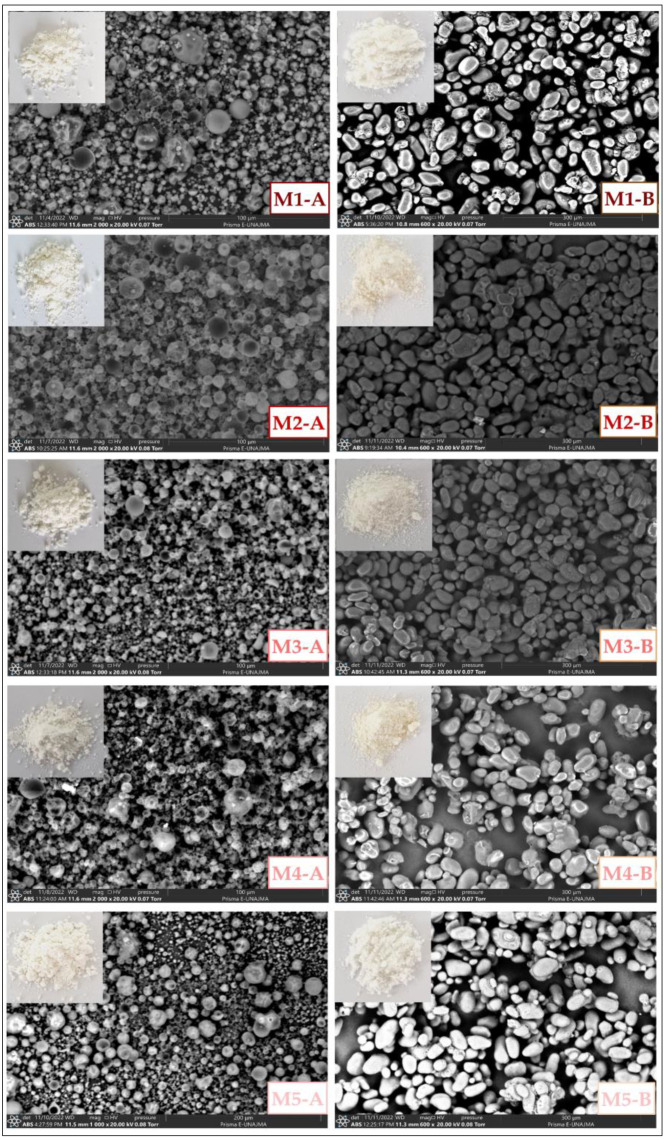
SEM images of microencapsulates A and B.

Figure 9 shows the SEM microphotographs of the microencapsulates; in the case of microencapsulates A, spheres of different sizes and smooth surfaces were observed, which in some cases presented indentations on their external part, which was produced with the increase of the evaporation temperature during spray drying. Similar microphotographs were reported by other authors who used maltodextrin and gum as encapsulants [11,45,59,60]. On the other hand, in microencapsulates B, elliptical particles of heterogeneous size and smooth surface were obtained. This continuous coating would indicate that the encapsulant with OSA starch is a better wall material since it allows better core protection [61,62].

The results of the surface elemental analysis by SEM-EDS are shown in Table 2. In the case of microencapsulates A, the carbon content varied between 48.4 and 75%, and that of oxygen was between 25 and 51.6%. On the other hand, in microencapsulates B, the carbon content varied between 40.1 and 46.3%, and the oxygen content between 53.7 and 59.9%. The values obtained in both cases corroborated the encapsulation of the propolis-honey mixtures due to the majority of content of carbon and oxygen atoms, which are chemical elements in the biopolymers used as wall materials [2,63,64].

#### 3.4.4. Analysis of Particle Size and Potential ζ

**Figure 10:**
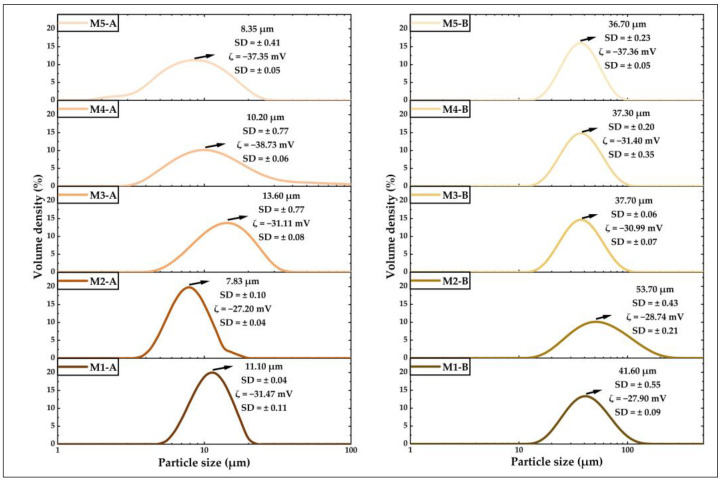
Particle size and potential ζ in microencapsulates A and B.

All the values obtained are shown in Figure 10. In the case of the particle size of microencapsulates A, values between 7.83 and13.6 µm were obtained, and in microencapsulates B, between 36.7 and 53.7 µm, observing that the latter were more prominent due to the wall material used (OSA starch/tara gum) and also to the structural changes that occurred in the spray drying process, which originated in the formation of stable heterogeneous microcapsules rich in proteins, fats, and glucides [44,65,66]. Regarding the results of the ζ potential, microencapsulates A reported values between −27.20 and −38.73 mV and microencapsulates B between −27.90 and −37.36 mV, which correspond to slight to medium stability in colloidal solutions [29].

#### 3.4.5. Thermal Analysis

**Figure 11:**
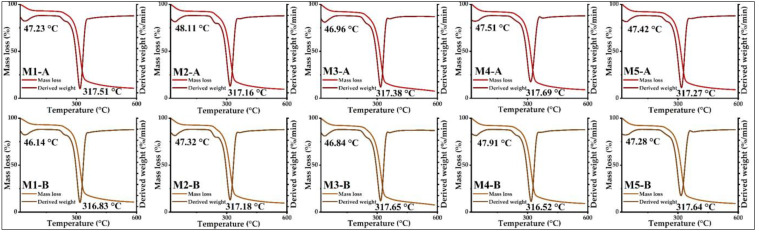
TGA and DTA curves for microencapsulates A and B.

The TGA and DTA curves of all the microencapsulates are shown in Figure 11, in which it can be appreciated that samples A and B had similar thermal behaviors for a temperature ramp from 20 to 600 °C; in the mentioned curves, it can be clearly appreciated the appearance of two main events. The first occurred between 46.84 and 48.11 °C with mass losses of approximately 5%, which is attributed to the breaking of hydrogen bridges and evaporation of water; also, other thermolabile compounds of low molecular weight were eliminated [2,36,67]. The second event was between 316.52 and 319.69 °C with mass losses of about 53% due to eliminating proteins, fats, carbohydrates, and other organic compounds of higher molecular weight. The decomposition of phenolic compounds and free amino acids occurs at temperatures above 200 °C due to the interaction of polyphenols with the matrices used in encapsulation [2,36,68]. At higher temperatures, the other organic compounds in the microencapsulates are eliminated, which continues until the final residues are obtained [2,57,59,64,69].

#### 3.4.6. Analysis by FTIR

**Figure 12:**
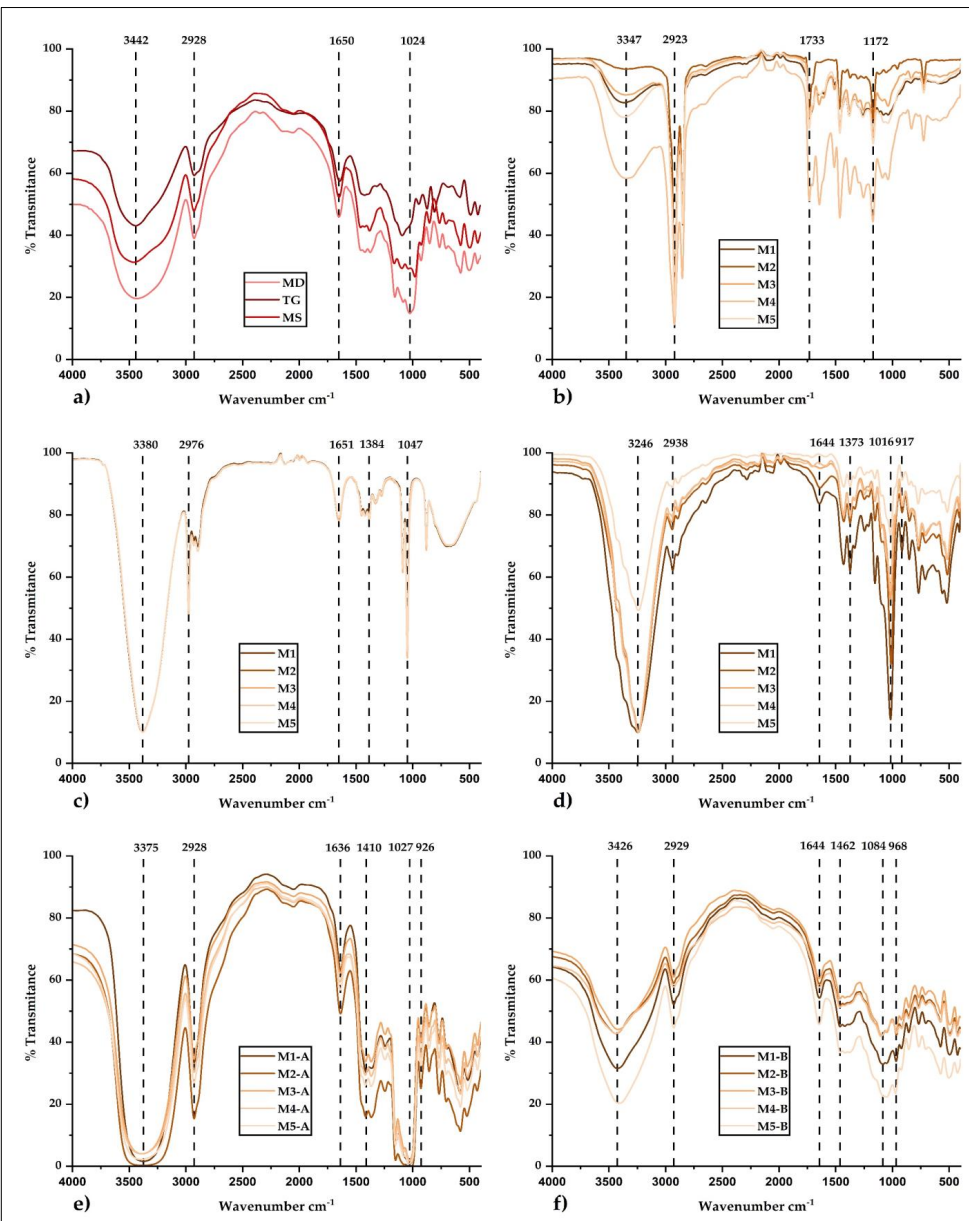
IR spectra of (**a**) wall materials, (**b**) crude propolis, (**c**) ethanolic extracts of propolis, (**d**) honey samples, (**e**) microencapsulates A, and (**f**) microencapsulates B.

Figure 12a shows the infrared (IR) spectra of the matrices used, Figure 12b,c the IR spectra of the crude propolis and ethanolic propolis extracts, and Figure 12d the IR spectra of the honey samples. In these figures, both core and wall materials contributed various functional groups to microencapsulates A and B (Figure 12e,f) [70]. Validated methods were used to perform the FTIR interpretation, which confirmed the cores’ encapsulation in the studied matrices due to the molecular structure obtained in the IR spectra [2,71]. In that sense, stretching stress bands between 3246–3442 cm^−1^ belonging to the hydroxyl and amino groups were observed in cores and encapsulants [38,72], indicating the presence of phenolic compounds, carbohydrates, proteins, and water [73,74].

The wavenumbers of 2928 and 2929 cm^−1^ present in microencapsulates A and B would correspond to the CH and NH3 functional groups belonging to carboxylic acids and amino acids. The 1636 and 1644 cm^−1^ voltage bands would correspond to carbonyl and ketone functional groups belonging to lipids, phenolic compounds, and flavonoids [75,76]. The wavenumbers of 1027 and 1084 cm^−1^ would correspond to the chemical groups ether, ester, alcohol, and carboxylic acid, belonging to ethanolic extracts of propolis and bee honey, which would be related in turn to the presence of different polyphenols and flavonoids [77,78]. The spectral region below the wavenumbers of 927 and 968 cm^−1^ would be related to the C-H functional group present in the aromatic rings of phenolic compounds [79]. Finally, the smaller peaks would correspond to the structural modifications suffered by the aromatic rings of the compounds present in microencapsulates A and B [80]. The aforementioned coincides with the reports for encapsulations of ethanolic extracts of propolis obtained by different procedures [36,38,81].

#### 3.4.7. Analysis by X-ray Diffraction (XRD)

**Figure 13:**
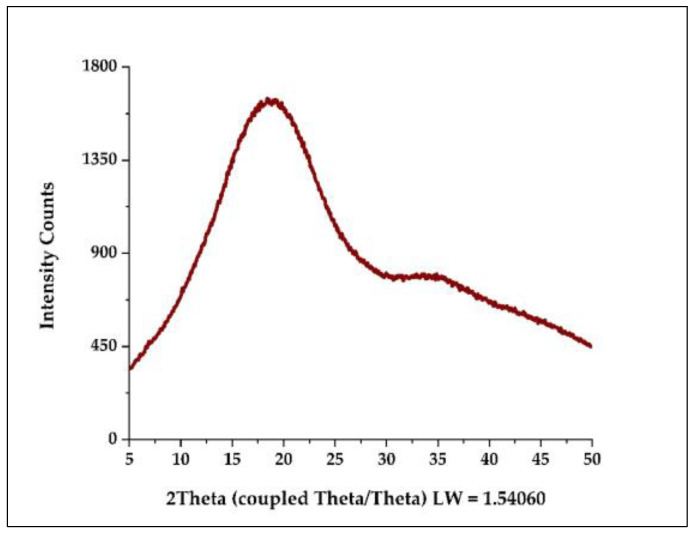
Typical XRD diffraction pattern in microencapsulates A and B.

The typical XRD pattern in microencapsulates A and B is shown in Figure 13, in which low crystallinity was observed, with a peak between 15 and 20° and slight diffraction between 33 and 37°, which would be attributable to the irregular size of the microparticles obtained. This behavior would indicate that these are amorphous samples and that the initial properties were not altered because the cores were molecularly dispersed in the encapsulants or that the proportions used were insufficient to modify the material’s properties [2,36,38].

#### 3.4.8. Kinetic Study of Phenolic Compounds

**Figure 14:**
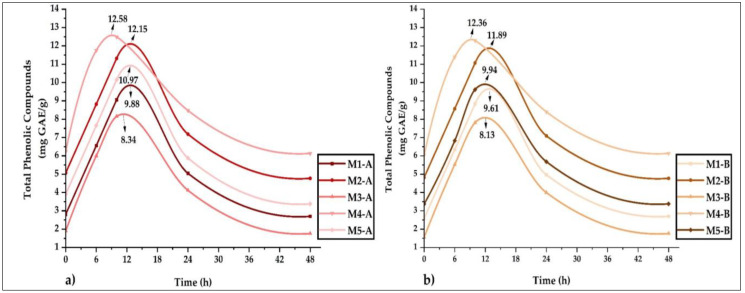
(**a**) Stability and release of microencapsulates A and (**b**) stability and release of microencapsulates B.

Figure 14a shows the release profiles for microencapsulates A, noting that the maximum values of polyphenols released were between 8.34 and 12.58 mg GAE/g. Figure 14b shows the curves obtained for microencapsulates B with values between 8.13 and 12.36 mg GAE/g. In both cases, it was observed that the maximum release time was between 7 and 13 h, and it was also noted that the highest release occurred in samples M4-A and M4-B. It is essential to highlight that microencapsulation B presented better protection of the bioactive compounds during release, information that is useful because microencapsulates could be used as additives in aqueous phase food products. On the other hand, the results obtained are useful to predict a possible similar behavior at the gastrointestinal level when products with these microparticles as ingredients are consumed [2,11,82].

Likewise, polyphenol release tests were performed at pH 3, 4, 5, and 6 on microencapsulates A and B; however, no changes were obtained that would allow reporting kinetic curves, which coincided with the results obtained in other similar research works [2,11].

#### 3.4.9. Particle Size in Aqueous Solution

The particle sizes in the aqueous solution in microencapsulates A varied between 13.06 and 250.3 nm and in the case of microencapsulates B between 1.44 and 430.8 nm, showing that they were partially dissolved in water (Table 3). The dynamic light scattering (DLS) technique was used to measure the behavior of the microencapsulates in the aqueous phase since they will preferably be used in liquid products of the food and pharmaceutical industry [2,55]. The sizes were similar to the values reported for encapsulates obtained by spray-drying ethanolic propolis extracts in maltodextrin and gum arabic [2].

## 4. Conclusions

High levels of polyphenols, flavonoids, and antioxidant activity were obtained in microcapsules of a mixture of propolis and honey when maltodextrin/tara gum and modified native potato starch/tara gum were used as wall materials during spray drying. Results showed good encapsulation efficiency, good solubility in cold water, low moisture levels, hygroscopicity, water activity, and bulk density typical of spray-drying processes. Instrumental analyses confirmed the encapsulation of the core in the studied encapsulants, the microcapsules obtained were tiny, with sizes ranging from 7.83 to 53.7 µm, which depended on the wall material used. Spherical and elliptical shapes, light and medium stability at ζ potential, typical chemical groups, and amorphous behavior were observed in the microparticles.

Modified native potato starch has been shown to be a better wall material than maltodextrin; both combine very well with tara gum in the microencapsulation of propolis and honey; stability and release studies of phenolic compounds showed high values between 7 and 13 h. The results obtained show that there is a potential for the use of microcapsules of propolis and honey as additives in the food and pharmaceutical industries.

## Figures and Tables

**Table 1:** Physical and chemical properties of microencapsulates A and B.

Microencapsulates A	M1-A		M2-A		M3-A		M4-A		M5-A	
**Properties**	$\overset{-}{x}\;$ ** ± SD**	*	$\overset{-}{x}\;$ **± SD**	*	$\overset{-}{x}\;$ **± SD**	*	$\overset{-}{x}\;$ **± SD**	*	$\overset{-}{x}\;$ **± SD**	*
Phenolic compounds	2.81 ± 0.01	a	5.07 ± 0.03	b	1.90 ± 0.03	c	6.24 ± 0.02	d	3.91 ± 0.01	e
Flavonoids	2.38 ± 0.05	a	5.14 ± 0.05	b	1.87 ± 0.05	c	6.71 ± 0.09	d	2.60 ± 0.05	e
DPPH	11.86 ± 0.30	a	18.66 ± 0.68	b	9.67 ± 0.39	c	22.19 ± 0.98	d	13.43 ± 0.39	e
ABTS	2.55 ± 0.06	a	3.13 ± 0.05	b	2.38 ± 0.06	c	3.64 ± 0.09	d	2.83 ± 0.05	e
Encapsulation efficiency (%)	29.45 ± 0.03	a	48.39 ± 0.16	b	25.57 ± 0.23	c	58.64 ± 0.11	d	40.08 ± 0.05	e
Yield (%)	51.23 ± 1.05	a	52.34 ± 0.81	ab	51.34 ± 1.20	a	53.24 ±0.89	a	52.25 ± 1.00	ab
Hygroscopicity (%)	8.34 ± 0.66	a	6.82 ± 0.06	b	8.22 ± 0.18	a	6.63 ± 0.18	b	6.94 ± 0.16	b
Solubility	90.28 ± 0.46	ab	90.59 ± 1.29	ab	91.26 ± 0.56	b	89.63 ± 0.87	a	92.84 ± 0.46	c
Moisture (%)	5.41 ± 0.03	a	6.87 ± 0.04	b	7.33 ± 0.06	c	7.23 ± 0.06	c	6.75 ± 0.09	d
Aw	0.26 ± 0.002	a	0.33 ± 0.001	b	0.36 ± 0.001	c	0.35 ± 0.001	d	0.33 ± 0.001	b
Bulk density	0.41 ± 0.02	a	0.33 ± 0.01	b	0.31 ± 0.01	b	0.33 ± 0.01	b	0.33 ± 0.01	b
L	92.39 ± 0.02	a	90.81 ± 0.04	b	92.16 ± 0.01	c	91.94 ± 0.08	d	92.82 ± 0.01	e
a	−0.78 ± 0.01	a	−0.46 ± 0.01	b	−0.85 ± 0.01	c	−1.12 ± 0.01	d	−0.88 ± 0.01	e
b	5.88 ± 0.01	a	7.19 ± 0.02	b	6.30 ± 0.01	c	6.56 ± 0.03	d	6.05 ± 0.05	e
**Microencapsulates** **B**	**M1-B**		**M2-B**		**M3-B**		**M4-B**		**M5-B**	
**Properties**	$\overset{-}{x}\;$ ** ± SD**	*	$\overset{-}{x}\;$ **± SD**	*	$\overset{-}{x}\;$ **± SD**	*	$\overset{-}{x}\;$ **± SD**	*	$\overset{-}{x}\;$ **± SD**	*
Phenolic compounds	2.59 ± 0.08	a	4.82 ± 0.03	b	1.56 ± 0.04	c	6.04 ± 0.02	d	3.37 ± 0.01	e
Flavonoids	2.24 ± 0.05	a	4.97 ± 0.03	b	1.77 ± 0.07	c	6.53 ± 0.09	d	2.45 ± 0.07	e
DPPH	11.79 ± 0.20	a	16.74 ± 0.49	b	8.71 ± 0.39	c	20.25 ± 0.59	d	12.81 ± 0.30	e
ABTS	2.27 ± 0.05	a	2.85 ± 0.03	b	2.05 ± 0.07	c	3.27 ± 0.09	d	2.67 ± 0.07	e
Encapsulation efficiency (%)	24.66 ± 0.39	a	46.01 ±0.16	b	20.93 ± 0.28	c	56.74 ± 0.09	d	34.46 ± 0.07	e
Yield (%)	55.83 ± 0.93	ab	57.03 ± 0.21	b	55.00 ±0.85	a	56.28 ± 0.45	ab	55.90 ± 0.85	ab
Hygroscopicity (%)	9.45 ± 0.16	a	7.93 ± 0.17	b	9.63 ± 0.19	a	7.61 ± 0.06	b	8.22 ± 0.18	b
Solubility	82.89 ± 1.46	a	81.83 ± 1.17	a	78.16 ± 0.57	b	76.14 ± 1.15	c	78.62 ± 0.96	b
Moisture (%)	5.28 ± 0.03	a	6.75 ± 0.05	b	7.03 ± 0.11	c	7.02 ± 0.10	c	5.29 ± 0.09	a
Aw	0.26 ± 0.001	a	0.33 ± 0.001	b	0.34 ± 0.001	c	0.34 ±0.002	c	0.26 ± 0.002	a
Bulk density	0.39 ± 0.01	a	0.32 ± 0.01	b	0.32 ± 0.01	b	0.32 ± 0.02	b	0.41 ± 0.02	a
L	92.00 ± 0.01	a	91.13 ± 0.01	b	92.26 ± 0.01	c	92.03 ± 0.01	d	92.56 ± 0.03	e
a	−0.76 ± 0.01	a	−0.48 ± 0.01	b	−0.73 ± 0.01	c	−1.19 ± 0.01	d	−0.94 ± 0.02	e
b	6.44 ± 0.01	a	7.34 ± 0.01	b	5.72 ± 0.01	c	6.46 ± 0.02	a	6.30 ± 0.01	d

Where: M1, M2, M3, M4, and M5 are the locations from which propolis and honey were obtained; $\overset{-}{x}$, arithmetic mean; SD, standard deviation. * Different letters per row indicate significant differences.

**Table 2:** Surface analysis of microencapsulates A and B by EDS.

Microencapsulate A	M1-A		M2-A		M3-A		M4-A		M5-A	
Element	C	O	C	O	C	O	C	O	C	O
Atomic %	64.7	35.3	48.4	51.6	75.0	25.0	71.5	28.5	51.4	48.6
Atomic % Error	0.1	0.1	0.1	0.2	0.2	0.1	0.2	0.1	0.1	0.1
Weight %	57.9	42.1	41.3	58.7	69.2	30.8	65.4	34.6	44.2	55.8
Weight % Error	0.1	0.1	0.1	0.3	0.1	0.1	0.1	0.1	0.1	0.2
**Microencapsulate B**	**M1-B**		**M2-B**		**M3-B**		**M4-B**		**M5-B**	
Element	C	O	C	O	C	O	C	O	C	O
Atomic %	46.3	53.7	40.1	59.9	41.0	59.0	41.5	58.5	41.0	59.0
Atomic % Error	0.1	0.2	0.1	0.2	0.1	0.2	0.1	0.2	0.1	0.2
Weight %	39.2	60.8	33.4	66.6	34.3	65.7	34.8	65.2	34.3	65.7
Weight % Error	0.1	0.2	0.1	0.2	0.1	0.2	0.1	0.2	0.1	0.2

Where: C: Carbon and O: Oxygen.

**Table 3:** Particle size in aqueous solution.

Microencapsulates	Peak	Size (nm)	%	Microencapsulates	Peak	Size (nm)	%
M1-A	1	219.30	100	M1-B	1	232.00	100
M2-A	1	250.30	100	M2-B	1	169.90	100
M3-A	1	168.30	89.53	M3-B	1	430.80	100
	2	13.06	10.47	M4-B	1	191.60	100
M4-A	1	174.80	100	M5-B	1	135.00	94.57
M5-A	1	153.9	100		2	1.44	5.43

Where: M1-A, M2-A, M3-A, M4-A, and M5-A are the microencapsulates A and M1-B, M2-B, M3-B, M4-B, and M5-B are the microencapsulates B.

## Data Availability

They are available in the same article.
